# Negative regulator NLRC3: Its potential role and regulatory mechanism in immune response and immune-related diseases

**DOI:** 10.3389/fimmu.2022.1012459

**Published:** 2022-10-20

**Authors:** Deyi Sun, Jiqian Xu, Wanying Zhang, Chaoying Song, Chenggang Gao, Yajun He, You Shang

**Affiliations:** ^1^ Department of Critical Care Medicine, Union Hospital, Tongji Medical College, Huazhong University of Science and Technology, Wuhan, China; ^2^ Institute of Anesthesiology and Critical Care Medicine, Union Hospital, Tongji Medical College, Huazhong University of Science and Technology, Wuhan, China

**Keywords:** NLRC3, NOD-like receptor, NF-κB, sting, pyroptosis, PI3K, immune response, immune-related diseases

## Abstract

NLRC3 is a member of the pattern recognition receptors nucleotide-binding oligomerization domain (NOD)-like receptors (NLRs) family, and plays a pivotal regulatory role in modulating the activation of immune cells. In macrophages, NLRC3 inhibits the activation of the NF-κB signaling pathway, the STING/TBK1 signaling pathway, and the formation of the inflammasome. In the context of T cells immune response, NLRC3 prevents the activation of T cells by regulating the function of dendritic cells and directly influencing the function of T cells. Different from other pattern recognition receptors, NLRC3 is more closely associated with regulatory activity than pathogens recognition, it influences the fates of cells, for example, prevents proliferation, promotes apoptosis and inhibits pyroptosis. These cellular functions regulated by NLRC3 are involved in the development processes of a variety of diseases, such as infectious disease, sterile inflammatory diseases, and cancer. However, its characteristics, function and regulatory mechanism in immune response and immune-related diseases have not been addressed fully. In this review, we elaborate the potential roles of NLRC3 from several different levels, include molecular mechanism, cellular functions in the immune-related diseases.

## 1 Introduction

NLRC3 is one of the members of NLR family. NLRs belong to the pattern recognition receptors (PRRs) family, which includes five sub-families, the Toll-like receptors (TLRs), C-type lectin receptors (CLRs), Rig-I-like helicase receptors (RLRs), nucleotide-binding oligomerization domain (NOD)-like receptors (NLRs), and absent-in-melanoma (AIM)-like receptors (ALRs) (1). TLR and CLR are membrane-bound receptors (2, 3), while NLR, RLR, and ALR localize to the cytoplasm (4–6). In general, once they recognize pathogen-associated molecular patterns (PAMPs) or damage-associated molecular patterns (DAMPs), the PRRs trigger a set of inflammatory signal cascades. Interestingly, plenty of evidence has demonstrated that NLRs exerted many other functions in addition to their pattern recognition role during the immune response (7–9).

The NLR family comprises 22 kinds of protein in human and 34 in mice. NLRs are mainly composed of three domains, a series of leucine rich repeats (LRRs) form the C-terminal domain, which is responsible for the ligand recognizing. The intermediate domain nucleotide binding/oligomerization domain (NBD) is responsible for self-oligomerization and some regulatory activities. The N-terminal domains of different NLR subfamilies also differ, such as PYRIN domain (PYD), caspase activation and recruitment domain (CARD), and acidic transactivation or a baculoviral inhibitory repeats (BIR) domain. The N-terminal domain of NLRs serve as a bridge to link the NLRs with downstream effector molecules or adopter proteins (10).

**Figure 1:**
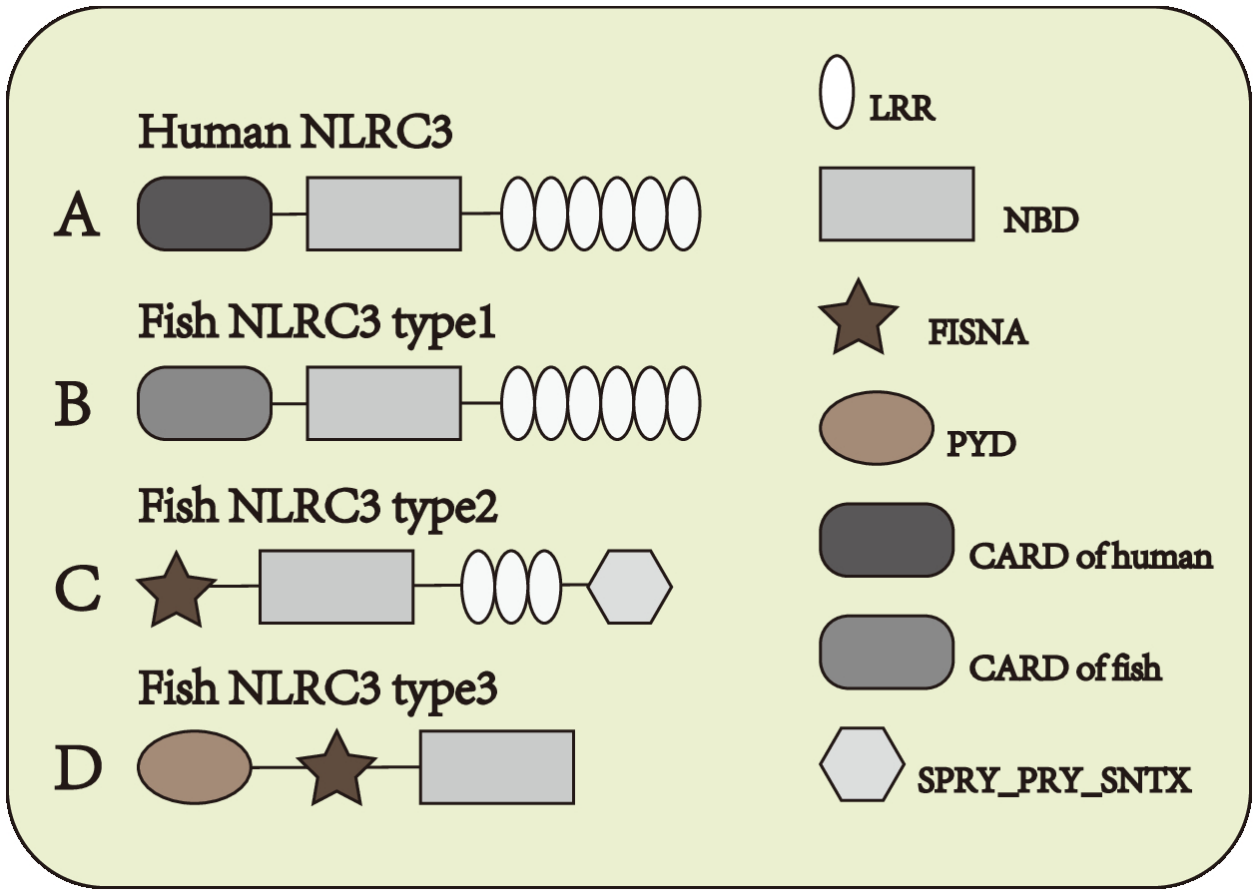
Structure of NLRC3 in different species. **(A)** From N-terminal to C-terminal, human NLRC3 is composed of CARD domain, NBD domain and LRR domain. **(B–D)** Three types of structures of fish NLRC3, from N-terminal to C-terminal, the composition and order of the domain are **(B)** CARD domain, NBD domain and LRR domain **(C)** FISNA domain, NBD domain, LRR domain and SPRY_PRY_SNTX domain **(D)** PYD domain, FISNA domain and NBD domain.

NOD-like receptor (NLR) family CARD domain containing-3 (NLRC3) is one of the members of NLRs which serves as a negatively regulatory molecule in cytoplasm. NLRC3, also known as CLR16.2 or NOD3, was first detected by Conti et al. (11) in human T lymphocytes in 2005. NLRC3 mainly expressed in immune system, and the expression of NLRC3 is highest in CD4+ and CD8+ T cells, with moderate expression in the thymus, lymph node, and other tissues of the immune system (11). And NLRC3 also can be detected in other issues, in some immune-related diseases also observed the expression changes of NLRC3. Under both stimulated and unstimulated states, NLRC3 is only detected in the cytoplasm (11). In mammals, the N-terminal domain of NLRC3 is composed by 6-α-helical bundle, such constructure is similarly to CARD domain and PYD domain, but it doesn’t quite fit the definition of either domain. Subsequently, the N-terminal domain of NLRC3 was defined as the CARD domain (12) **Figure 1A**, different from other NLRs such as NLRX1, NLRC4, and NOD2, the LRR domain of NLRC3 contains a series of continuous positive charges, which are responsible for the affinity between NLRC3 and ligands (13). In fish, the structure of NLRC3 also differs in different fish. It can be divided into three structures. Type 1: Composed of CARD domain, NBD domain and LRR domain **Figure 1B**, the CARD domain of type I-NLRC3 is closer to the CARD domain of the human NLRC family. Type 2: Composed of FISNA domain, NBD domain, LRR domain and PRY/SPRI domain **Figure 1C**, the LRR domain of type 2-NLRC3 is shorter than that of type 1. Type 3: Consists of PYD domain, FISNA domain, and NBD domain. **Figure 1D**

## 2 Roles of NLRC3: From the molecular biological perspective

### 2.1 Inhibition of the NF-κB signaling pathway

**Figure 2:**
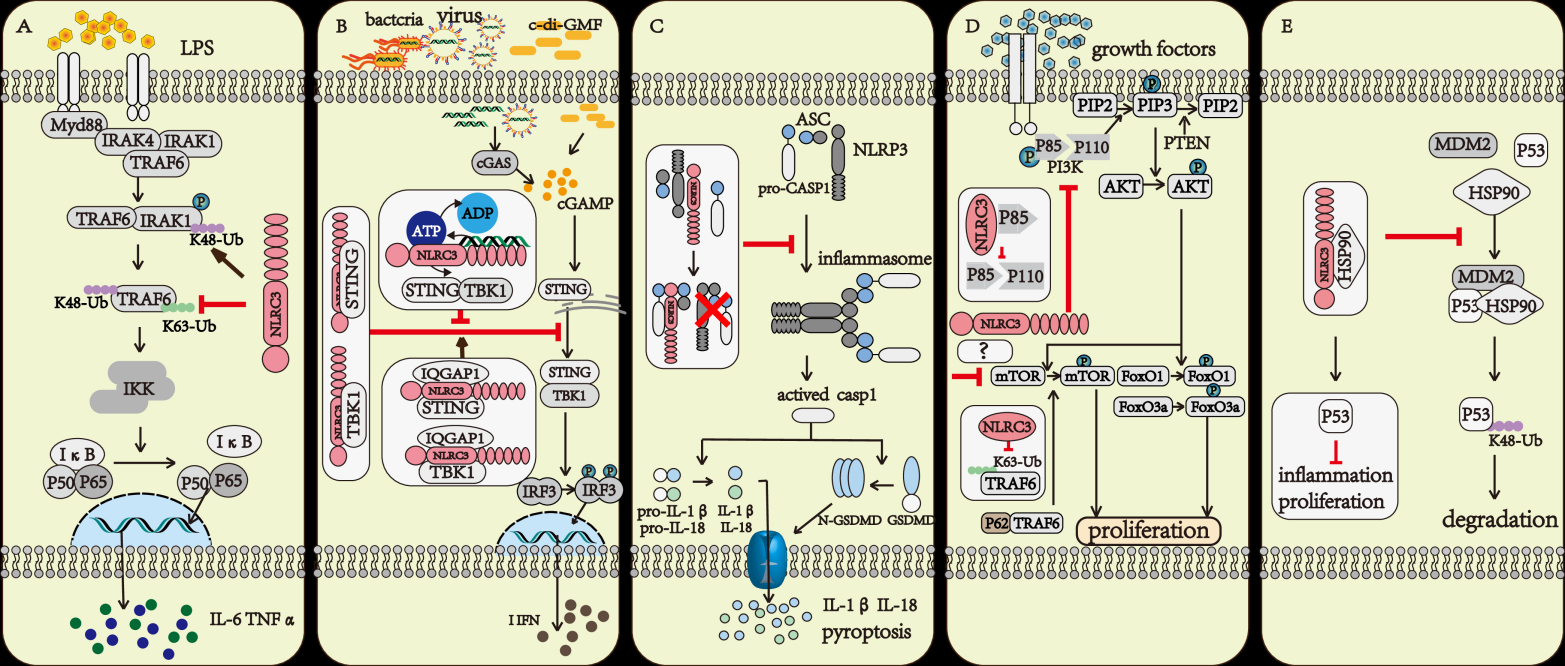
The regulatory mechanism of NLRC3. **(A)** The TRAF6 and IRAK1 activation upon LPS stimulation further activate the NF-κB signaling pathway. NLRC3 prevents the K63-linked polyubiquitination of TRAF6 and promotes the degradation of TRAF6 through proteasome, or NLRC3 promotes the K48-linked polyubiquitination of IRAK1 and promotes the degradation of IRAK1 through proteasome. **(B)** Upon the Bacteria, Virus infection and c-di-GMF stimulation, the DNA binding to LRR domain of NLRC3 increased the activation of ATPase on the NBD domain of NLRC3, prevented the association of NLRC3 and STING and inhibited the STING/TBK1 signaling pathway. IQGAP1 interacted with NLRC3, promoted NLRC3 to bind with STING and TBK1, and further enhanced the inhibitory effect of NLRC3 on STING/TBK1 signaling pathway. All further attenuates the production of type I interferon (I-IFN), preventing the inflammatory response of macrophages. **(C)** NLRC3 interacts with ASC and pro-CASP1 through the NBD domain, then prevents the formation of the inflammasome, and makes cells stay away from pyroptosis. **(D)** NLRC3 interacts with the p85 subunit of PI3K, then inhibits the interaction of p85 submit and catalytic submit p110α, preventing the phosphorylation and activation of the p110α submit, which blocks the transmission of downstream signals, including PI3K-AKT-mTOR and PI3K-AKT-FoxO3a/FoxO1 signaling pathways; P62 interacting with TRAF6 induced the activation of mTOR, NLRC3 prevents the K63-linked polyubiquitination of TRAF6, and negatively regulates the mTOR signaling pathway and prevents cell proliferation. **(E) **NLRC3 interacts with HSP90 to inhibit HSP90-mediated ubiquitination of P53 and inhibit the degradation of P53 in lysosomes.

The NF-κB signaling pathway is one of the most important inflammatory signaling pathways both in innate immunity and adaptive immunity (14). While macrophages were stimulated by LPS, the NF-κB signaling pathway was activated and a variety of proinflammatory cytokines such as IL-1, IL-6, and TNF-α release. If NLRC3 was overexpressed, the activation of the NF-κB signaling pathway was inhibited. The result of co-immunoprecipitation indicated that NLRC3 interacted with TRAF6, decreased the K63-linked ubiquitination of TRAF6, and remained the K48-linked ubiquitination on it. As a result, NLRC3 not only inhibited the activation of TRAF6, but also promoted the degradation of TRAF6 through the proteasome, further reduced the abundance of TRAF6 in cytoplasm (14). Moreover, NLRC3 also negatively regulates the NF-κB signaling pathway by regulating the abundance of IRAK1 (15). NLRC3 promotes the K48-linked ubiquitination on interleukin-1 receptor-associated kinase1 (IRAK1), which decrease the abundance of IRAK1 through the proteosome (15) (**Figure 2A**).

### 2.2 Disrupting the STING/TBK1 signaling pathway

The stimulator of interferon gene (STING) is an intracellular DNA sensor that regulates the production of type I interferon (I-IFN) by interacting with TANK-binding kinase (TBK1) (16). When stimulated by cytosolic DNA, cyclic di-GMP (c-di-GMP), and DNA viruses, the immune response was up-regulated in NLRC3 deficient mouse embryonic fibroblasts (MEFs) and bone-marrow-derived macrophages (BMDMs). The result of co-immunoprecipitation indicated that NLRC3 prevented the interaction between STING and TBK1 *via* associating with both STING and TBK1 through its NBD domain. Furthermore, NLRC3 also prevented the trafficking of STING to the perinuclear and punctate region, which is important for regulating the production of I-IFN (16). During viral infection, TLR9 and STING activated the NF-κB signaling pathway, which attenuated the expression of NLRC3. This regulatory effect can be considered as a positive feedback loop that amplifies the immune response after infection (16, 17) (**Figure 2B**).

When regarding to the regulation of NLRC3, there has also been some progress in recent years (13, 18). Viral DNA associated with the LRR domain of NLRC3, and increased the ATPase activity of the NBD domain of NLRC3, further promoted the release of STING from NLRC3, and in the end, activated the STING/TBK1 signaling pathway (13). IQGAP1 is a famous actin- and tubulin-binding protein (18). In macrophages, IQGAP1 interacts with the actin nucleating protein, diaphanous-related formin (Dia1), to take part in phagocytosis and phagocytic cup formation during infection. The rearrangement of the cytoskeleton is important for the phagocytosis activity of macrophages (19, 20). Tocker et al. (18) demonstrated the interaction between the RGCT domain of IQGAP1 and the NBD domain of NLRC3 using yeast two-hybrid screening and coimmunoprecipitation experiments in human cell lines. And then they found in the following experiment that the deficiency of IQGAP1 promoted the activation of the I-IFN signaling pathway. Thus, together with previous evidence (16), the authors speculated that IQGAP1 interacts with NLRC3 to inhibit the STING-TBK1 signaling pathway, which in turn attenuates the production of I-IFN (18) (**Figure 2B**).

### 2.3 Preventing the formation of the inflammasome and cell pyroptosis

The inflammasome-forming subfamily of NLRs (such as NLRP3, NLRP1, NLRC4) recognize PAMP or DAMP or the changes in the intracellular environment, then recruit homologous NLRs, the apoptosis-associated speck-like protein (ASC) and pro-caspase1 to form the inflammasome, further activate CASP1, lead to pyroptosis (21). Pyroptosis, a form of programmed necrosis, amplifies inflammation during the immune response. NLRC3 could inhibit cell pyroptosis by preventing the formation of the NLRP3- and NLRC4-inflammasomes (22).Through interacting with ASC and pro-caspase-1 *via* the CARD domain, NLRC3 prevented the interaction of ASC and pro-caspase-1, and disrupted the formation of the ASC speck, further inhibited cell pyroptosis (22) (**Figure 2C**).

### 2.4 Suppressing the PI3K signaling pathway

NLRC3 negatively regulated the PI3K-AKT-mTOR signaling pathway in mouse bone-marrow-derived macrophages during stimulated with insulin-like growth factors(IGF-1) or LPS (23). mTOR plays multiple roles during the life process of a cell such as regulating the survival, proliferation, metabolism, autophagy, and immunity of cells (24, 25). NLRC3 interacted with the p85 subunit of PI3K to prevent the interaction between the p85 submit and the p110α submit of PI3K, further inhibited the phosphorylation and activation of p110α PI3K and then blocked the transmission of downstream signals (23) (**Figure 2D**).

NLRC3 has also been reported to interact with TRAF6 and directly associate with mTOR further prevented the activity of mTOR (26). The signal adaptor P62 protein interacts with TRAF6 and promotes the translocation of mTOR to the lysosome and its subsequent activation (27). NLRC3 interacted with TRAF6 to promote its degradation *via* the proteasome (14). In 239T cells with NLRC3, TRAF6 and mTOR overexpressed, researchers observed that NLRC3 interacted with both TRAF6 and mTOR (26). Thus, we can speculate that NLRC3 interacts with TRAF6 and promotes the degradation of TRAF6 to prevent TRAF6 interaction with p62 and further prevents the activation of mTOR. NLRC3 could also directly associate with mTOR to prevent its activity, and the specific mechanisms underlying this inhibition requires to be further studied (**Figure 2D**).

The interaction between NLRC3 and the p85 submit of PI3K not only has effect on the PI3K/AKT/mTOR signal axis, but also on PI3K/AKT/FoxO3a or FoxO1 signaling pathways (26). FoxO3a and FoxO1 contribute to regulate cell proliferation, apoptosis, metabolism, and survival (28). AKT activates and prevents ubiquitin-mediated degradation of FoxO3a and FoxO1 (29). In colonic tissue of NLRC3 ablated mice, the activation of AKT and phosphorylation of FoxO3a and FoxO1 are higher than in WT mice. NLRC3 ablated colonic epithelial cells exhibit an increase in cellular proliferation (26) (**Figure 2D**).

### 2.5 Preventing the degradation of P53

Tumor suppressor protein P53 inhibits tumor growth in various ways and plays an important role in maintaining genomic stability and preventing abnormal cell proliferation. Ubiquitination of P53 is an important way to regulate this protein (30). NLRC3 associates with HSP90(Heat Shock Protein 90), preventing the interaction between HSP90, MDM2 and P53, further inhibiting the ubiquitin and degradation of P53 (31) (**Figure 2E**).

### 2.6 Interfering with the MAPK signal pathway

The family members of the mitogen-activated protein (MAP) kinases mediate a wide variety of cellular behaviors in response to extracellular stimuli. MAPKs are divided into four main signal pathways, P38 signal pathway is one of them, which is mainly related to the production of inflammatory cytokines and cell apoptosis (32). In DCs isolated from NLRC3 knockout mice, researchers observed that the phosphorylation of P38 and the activation of its downstream signaling pathway were up-regulated than WT (8). However, the specific mechanisms through which NLRC3 regulates the activation of the MAPK signaling pathway are still unclear and further study is needed (8).

## 3 Roles of NLRC3: The influence on cell function

### 3.1 The regulatory effect of NLRC3 on cell fate

#### 3.1.1 NLRC3 inhibit cell proliferation

**Figure 3:**
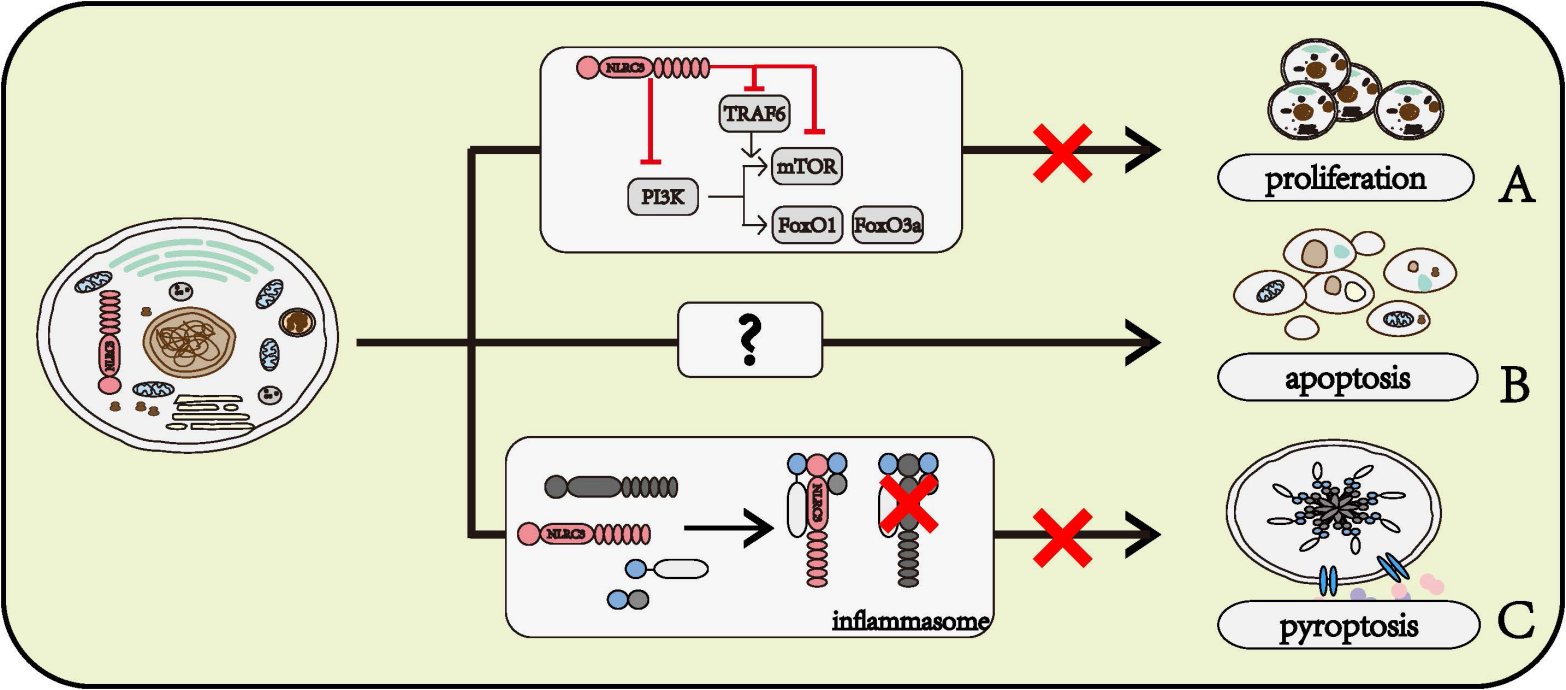
Roles of NLRC3 on cell fates. **(A)** NLRC3 inhibits cell proliferation. NLRC3 interferes the normal function of PI3K or inhibits the activation of mTOR through directly regulating itself and interfering TRAF6, thereby preventing its downstream molecules mTOR, FoxO1 and FoxO3a, and further regulate the cell proliferation. **(B)** NLRC3 might promote the cell apoptosis. **(C)** NLRC3 prevents the interaction with ASC and pro-CASP1, further inhibit the formation of inflammasome and cell pyroptosis.

The PI3K signaling pathways play an important part in the regulation of cell proliferation. NLRC3 prevented the interaction between P85 and P110α submits of PI3K, further inhibited the activity of mTOR and FoxO1/FoxO3a, which act as regulators of cell proliferation (23). NLRC3 also disrupted the function of mTOR through preventing the interaction between TRAF6 and P62, and NLRC3 directly interacted with mTOR to inhibit the activation of mTOR (26). Given the amount of research data and the strong research evidence, the role of NLRC3 in regulating cell proliferation is beyond all doubt. **Figure 3A**

#### 3.1.2 NLRC3 promotes cell apoptosis

In the AOM-DSS model of colorectal tumorigenesis, the activation of CASP8, CASP3, and CASP7 was lower in the colon of NLRC3 knockout mice than in WT mice (26). Flow cytometry phenotyping and TUNEL assay findings indicated the apoptosis rate of NLRC3 siRNA-treated HuH‐7 cells, a human liver cancer cell line, was significantly lower than in the scramble-siRNA-treated group after IL‐6 stimulation (33). Based on these data, we can speculate that NLRC3 has a role of promoting cell apoptosis, however, the mechanism by which NLRC3 promotes cell apoptosis remains to be determined (**Figure 3B**)

#### 3.1.3 NLRC3 prevents cell pyroptosis

The pyroptosis of THP-1 has been observed to be inhibited by NLRC3 (22). NLRC3 interacts with pro-caspase1 and ASC through CARD domain, prevents the formation of inflammasome, further inhibits cell pyroptosis (22) (**Figure 3C**).

### 3.2 The regulatory effect of NLRC3 on immune function

#### 3.2.1 Macrophage

**Figure 4:**
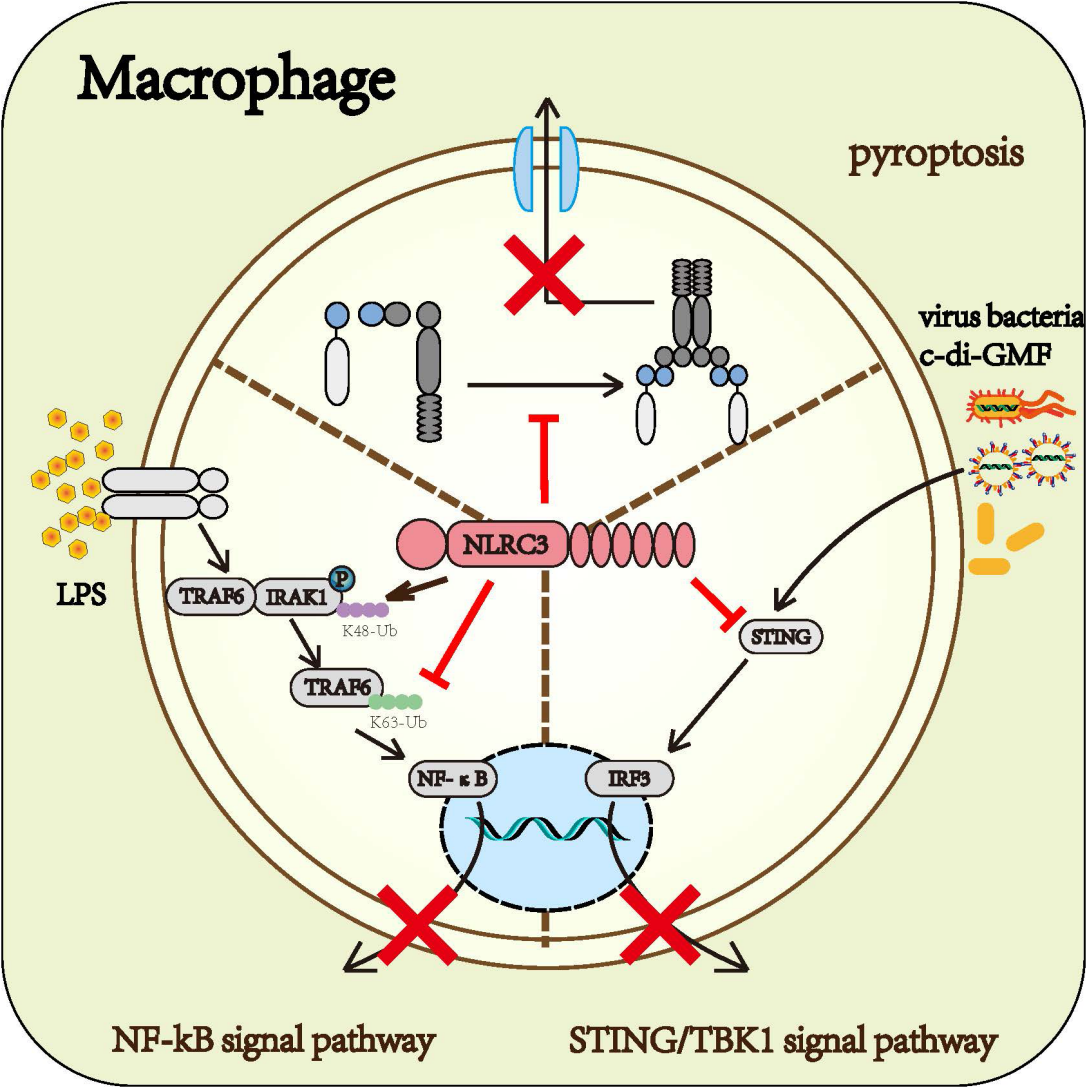
Roles of NLRC3 on macrophage: Upon the infection of gram-negative bacteria, NLRC3 decreases the activation of the NF-κB signal pathway and the release of multiple inflammatory cytokines such as TNF-α and IL-6 by TRAF6-dependent manner. Upon the infection of the virus, NLRC3 prevents the activation of STING/TBK1 signal pathway and the following release of I-IFN through inhibiting STING and TBK1 from functioning normally. Upon the PAMPs stimulation, NLRC3 competitively binding ASC and pro-CASP1 with NLRP3 prevents the formation of inflammasome and pyroptosis.

Macrophage is one of the most potent cells of the innate immune system. Based on existing research, it can be concluded that NLRC3 prevents the activation of macrophage. NLRC3 negatively regulated the NF-κB signaling pathway (14, 15), STING/TBK1 signaling (16), and the formation of the inflammasome (22), further prevented macrophages from producing inflammatory factors, thereby inhibited the macrophage immune response (**Figure 4**)

#### 3.2.2 Dendritic cell

**Figure 5:**
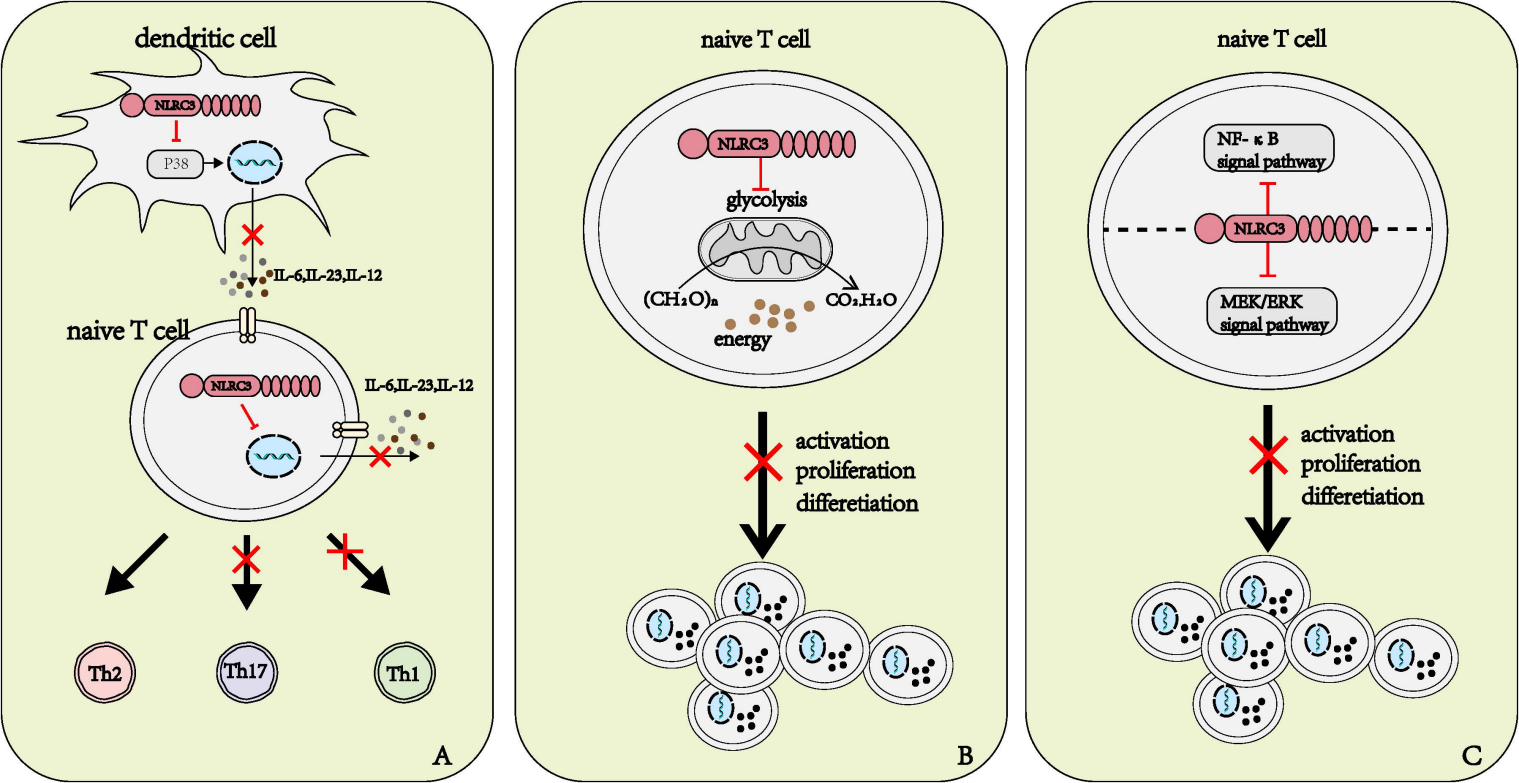
Roles of NLRC3 on T cells. **(A)** NLRC3 affects the differentiation direction of naïve T cells. NLRC3 prevents the production and release of IL-6, IL-23 and IL-12 in dendritic cells and naïve T cells, in DCs, NLRC3 executes regulatory roles by affecting P38 signal pathway. IL-12 promotes the differentiation of naïve T cells into Th1 cell, and IL-23, IL-6 promote the differentiation of naïve T cells into Th17 cell. **(B, C)** The potential several manners of NLRC3 affects the activation, proliferation and differentiation of T cells. **(B)** Sufficient energy is critical for the activation, proliferation and activation of naïve T cells, NLRC3 regulates the activation, proliferation and differentiation through negatively regulating mitochondrial function and the production of energy in naïve T cells **(C)** NLRC3 inhibits the activation of both NF-κB and MEK/ERK signal pathways and further inhibit the activation, proliferation and differentiation of T cells.

The dendritic cell (DC) is the most effective antigen-presenting cell among the immune system (34). Once stimulated by antigen, activated DCs increase the expression of surface co-stimulatory molecules and a series of cytokine to activate naïve T cells and promote their differentiation. Different cytokines secreted by DC induce different differentiation directions of naïve T cells. IL-12 promotes the differentiation towards Th1 cells, IL-4 promotes the differentiation towards Th2 cells (35); and IL-6, IL-23, and TGF-β promote the differentiation towards Th17 cells (8, 35, 36). NLRC3 can regulate the antigen-presenting function and the activity to activate and polarize CD4+ T cell Th1 and Th17 cells of DC (8). When NLRC3 was deficient in DCs, the researchers observed the production of IL-12, IL-6, and IL-23 were up-regulated. In the *in vivo* model, NLRC3 deficiency also led to the same result (8). Enhanced phosphorylation of P38 and the activation of downstream signaling pathway in DCs were observed in NLRC3 knockdown mice compared to DCs from wild-type (WT) mice. Thus, we can conclude that the regulatory role of NLRC3 is dependent on the P38 signaling pathway (**Figure 5A**). However, the specific mechanisms through which NLRC3 regulates the activation of P38 are still unclear and further study is needed (8).

#### 3.2.3 T lymphocytes

T cells are the most important members of the adaptive immune system. NLRC3 was first detected in T cells, and it is in T cells, thymus and lymph nodes where the expression of NLRC3 is the most abundance (11). Recently, NLRC3 was reported to negatively regulate the activation, differentiation, and proliferation of CD4+T cells in response to viruses (37), bacteria (38), and auto-antigen (37).

NLRC3 influences the polarization of T cells partly by regulating the production of cytokine. As mentioned above, NLRC3 influenced the cytokine produced by DCs to promote naïve T cell to polarize to Th1 and Th17 subsets (8). Recently, when researchers stimulated WT and NLRC3-deficient CD4+ naïve T cells with anti-CD3 and anti-CD28, they observed that some characteristic genes of Th1, Th17 and activated T cells up-regulated from the resulting heatmap of the RNA-seq expression analysis (37). We can speculate that NLRC3 affect the differentiation of naïve T cells not only by the influence on DCs, but *via* the direct regulatory effect on T cells. Further studies are needed to clarify the specific mechanisms of how NLRC3 regulates the differentiation of naïve T cells in a T-cell intrinsic manner (**Figure 5A**).

NLRC3 prevents the activation of T cells partly by influencing the metabolism of T cells. Metabolic alterations are highly correlated with T cell activation and differentiation (39). For instance, aerobic glycolysis is associated with the activation and the polarization to Th1 of T cells (40). Activated NLRC3-defincient CD4+T cells exhibited higher glycolytic capacity, glycolytic reserve, and maximal mitochondrial respiration than WT CD4+T cells (37). Such metabolic alterations provided the greater metabolic capacity to cope with sudden energy demands, in the other word, it provided energy for cell proliferation and cytokine production (37) (**Figure 5B**).

NLRC3 prevents the activation of T cells partly by regulating the NF-κB and MEK-ERK signal pathways (37, 38). Similar to macrophages, NLRC3 can negatively regulate the NF-κB signaling pathway by preventing the K63-linked polyubiquitin of TRAF6 in T cells (37). NLRC3 also inhibits the phosphorylation of ERK, but the specific manners by which NLRC3 influences the MEK-ERK signal pathway need further exploration (38). However, when using the inhibitors toward NF-κB or MEK-ERK signal pathways alone fail to prevent the activation of CD4+T cells with NLRC3 ablation. In a word, NLRC3 prevents the activation, proliferation and differentiation of T cells by inhibiting both NF-κB and MEK/ERK signal pathways (38) (**Figure 5C**).

## 4 Role of NLRC3 in immune-related diseases

### 4.1 NLRC3 in cancer

Inflammation plays important roles in the occurrence and development of cancer. Uncontrolled inflammatory response can be detected during several stages of tumor progression, include initiation, promotion, malignant conversion, invasion, and metastasis (41, 42). Abnormal cell proliferation is the hallmark of cancer; for many years, researchers have been working on the discovery and development of drugs that inhibit the abnormal cell proliferation in cancers (43, 44).

NLRC3 has been shown to inhibit cell proliferation, promote cell apoptosis, and prevent inflammation (8, 16, 23, 26, 37, 45). Thus, we can broadly speculate that NLRC3 may be a therapeutic target for tumors or can be used as an index to diagnosis or evaluate the prognosis of tumors. Recently, a large body of literatures have confirmed the beneficial role of NLRC3 in cancer treatment.

Several bioinformatics papers and some reports from detecting clinical specimens have demonstrated that NLRC3 is associated with the prognosis and malignancy of tumors (include colorectal cancer, Hepatocellular carcinoma, Gastric cancer, Bladder cancer and lung adenocarcinoma) (46–52).

The specific mechanism through which NLRC3 affect tumors have been reported as followed:

- *PI3K-AKT-mTOR and PI3K-AKT-FoxO3a/FoxO1 signal pathways* are associated with the suppressive effect of NLRC3 on colorectal cancer (23, 26).
- *JAK2/STAT3 signal pathway* is associated with the suppressive effect of NLRC3 on hepatocellular carcinoma (33).
- *Wnt/β-catenin signal pathway* is associated with the suppressive effect of NLRC3 on bladder cancer (46).
- *NLRC3 is involved in CD8+T infiltration during HCC, which may be associated with the increases of CCL5 (C-C motif chemokine ligand 5) as well as CXCL9 (C-X-C motif chemokine ligand 9)* (53).

### 4.2 NLRC3 in infectious diseases

#### 4.2.1 RNA virus

The regulatory role of NLRC3 in RNA virus infection has been mainly studied in adaptive immunity, that is, the effect of NLRC3 on CD4+T cell function. First of all, NLRC3 inhibits the body’s antiviral immunity. NLRC3 knockout mice showed a higher level of immune response, higher serum cytokine content, and relatively less viral load and pathological damage to organs (37, 54). In addition, NLRC3 regulates CD4+T cell function mildly, acting more like a rheostat than a switch (37).

#### 4.2.2 DNA virus

The regulatory effect of NLRC3 on the body’s anti-infection immunity to DNA viruses is mainly focused on innate immunity. NLRC3 inhibits the body’s immune response against DNA viruses (16). STING/TBK1 pathway plays an important role in the body’s anti-DNA virus immunity. NLRC3 interacts with STING and TBK1 to inhibit this signaling pathway (16), after recognizing viral DNA, STING interacts with TBK1 to promote IFN synthesis. In addition, viral DNA interacts with the LRR domain of NLRC3 to inhibit the binding between NLRC3 and STING, thereby activating the STING/TBK1 antiviral signaling pathway (13).

#### 4.2.3 Gram negative bacillus

As described above, NLRC3 inhibits the activation of NF-κB signaling pathway by interacting with TRAF6 and IRAK1 (14, 15). Keratitis caused by *Pseudomonas aeruginosa* (PA) is extremely common and damaging. PA can injure the corneal tissue *via* two mechanisms: bacteria invasion and excessive local inflammation (55). NLRC3 exerts beneficial roles in PA-induced keratitis (15).The expression of NLRC3 is decreased in mouse corneal tissue and macrophages cultured *in vitro* after PA infection. NLRC3 prevents the activation of macrophages and attenuates the cytokines release by macrophage through negatively regulating the NF-κB signaling pathway (15)

#### 4.2.4 Mycobacterium tuberculosis

NLRC3 mediates immune evasion of Mycobacterium tuberculosis *(M. tuberculosis)* after infection *in vivo* (38), while downregulation of NLRC3 expression has a protective effect on *M. tuberculosis* infection (38). *M. tuberculosis* is particularly devious that it can evade clearance by the host immune system. In general. macrophages engulf *M. tuberculosis* upon infection, but macrophages have poor bactericidal ability and carry the bacteria to the deep tissue without infection, in which forming the origin of tuberculosis granuloma. After that, chemokine binds to the chemokine receptor CCR22 of macrophages, and drives macrophages to accumulate and adhere to infected tissues, and finally form the tuberculous granuloma, with caseous necrosis of necrotic tissue and cells in the center, epithelioid cells in the periphery, and T cells and B cells in the periphery (56). Inhibiting the activation of CD4+T cells is one mechanisms of tuberculous granuloma formation (57). NLRC3 prevents the activation, proliferation, and differentiation of CD4+T cells by inhibiting the NF-κB and ERK/MEK signaling pathways, thereby attenuating the body’s anti-tuberculosis immunity, and the inhibition of CD4+T cells in turn suppresses the innate immune responses and further promotes *M. tuberculosis* survival (38).

### 4.3 NLRC3 in sterile inflammatory diseases

#### 4.3.1 Multiple sclerosis

Multiple sclerosis (MS) is a kind of chronic progressive inflammatory disease of the central nervous system (CNS) caused by autoimmune response. MS is characterized by the imbalance of the subsets of T cells, a large amount of Th1 and Th17 can be detected in CNS tissue, cerebrospinal fluid (CSF), and blood of patients with MS (58).

The overexpression of NLRC3 could improve the prognosis of MS (8, 37, 59–61). NLRC3 influenced the activation and polarization of the naïve T cells, and promoted the differentiation of naïve T cells into Th1 and Th17 cells (8, 37). Compared with WT mice, NLRC3 knockout mice were more likely to develop MS, and the researchers detected more infiltration of Th1 and Th17 cells in the spinal cord of NLRC3 knockout mice (37). Furthermore, data from RNA-seq studies indicated that the expression of NLRC3 is lower in CD4+T cells isolated from MS patients than in healthy volunteers (37). And when injected vaccines with NLRC3 overexpressing DCs, the MS progression in mice models was attenuated (8).

Epoxyeicosatrienoic acids (EETs) is generated during arachidonic acid metabolism and has the neuroprotective function (62). However, as soon as EET production, it is degraded by soluble epoxide hydrolase (sEH) (63). Previous research has confirmed that the sEH inhibitor TPPU has beneficial effects on neurodegenerative inflammatory diseases (64). Biliktu et al. reported that TPPU attenuated chronic MS development and the therapeutic effects of TPPU were associated with NLRC3 (61). In MS mice, TPPU increased the expression of NLRC3 in brain and spinal cord (61). Their finding was consistent with other researchers.

#### 4.3.2 Alzheimer’s disease

Alzheimer’s disease (AD) is chronic progressive neurodegenerative disease, and the formation of amyloid plaques and NFTs are the characters of AD. Amyloid plaques and NFTs activate microglia and astrocytes, lead to the release of large amounts of pro-inflammation cytokines and reactive oxygen species (ROS), and injure neurons (65).

NLRC3 has beneficial effects on AD (66).NLRC3 has been proved to be one of the most associative proteins of AD through an exome-wide rare variant analysis. The rare variants of NLRC3-coding gene had been observed in EMIF-AD (67). Overexpression of NLRC3 improved learning and memory ability in mouse, and NLRC3 inhibited the deposition of Aβ, the activation of glial cells and the degeneration of neurons in AD mouse (66). Overexpression of NLRC3 could attenuate the activation of PI3K in AD models *in vivo* or *in vitro*. And in AD mouse, researchers also detected NLRC3 in the nucleus of nerve cells (66). This is in contrast to previous reports that NLRC3 localized in cytoplasm both in physiological state and in response to LPS. The location of NLRC3 in neural nuclei may have contact with AD, which requires further investigation.

#### 4.3.3 Cerebral ischemia-reperfusion injury

One of the most important mechanisms of cerebral ischemia-reperfusion is neuroinflammation. A large amount of pro-inflammation factors result in neuronal cell death (68). Sevoflurane was observed to play beneficial roles on cerebral ischemia-reperfusion injury, and NLRC3 enhanced the beneficial effects of sevoflurane on ischemia-reperfusion models (28). Experimental evidence suggests that the beneficial effects of NLRC3 profited from the anti-inflammatory action *via* preventing NF-κB signal pathway (28).

#### 4.3.4 Cutaneous wound healing

Cutaneous wound healing is an intricate progress which is composed of hemostasis, inflammation, proliferation and remodel phases. And the initial inflammatory phase and the following proliferative phase are closely related to the outcome of cutaneous wound healing (69). NLRC3 prevents the inflammation and proliferation during cutaneous wound healing, NLRC3 interacts with Hsp90, inhibits the ubiquitin of P53, further promotes the inhibition on inflammation and cell proliferation by P53 signal pathway (31) and prevents cutaneous wound healing.

### 4.4 NLRC3 in other diseases

#### 4.4.1 Pulmonary hypertension

Pulmonary hypertension (PH) is a chronic progressive disease, It is the prominent feature of PH that pulmonary artery smooth muscle cells (PASMCs) release inflammatory cytokines and induce pulmonary vascular remodeling (70).

A randomized controlled trial first reported the relationship between NLRC3 and PH (71). The clinical study enrolled 43 patients with PH and 20 healthy controls. The results showed that the serum concentration of NLRC3 in patients with PH was lower than that in the control group, and the concentration of serum NLRC3 was inversely proportional to the severity of PH (71).

The animal experiment evidence suggested that NLRC3 inhibited the proliferation, migration, and inflammation of PASMCs and improved the symptoms of PH. Further, in the presence of agonists of PI3K-mTOR signal pathway, the beneficial effect of NLRC3 in the PH model were reversed (72, 73). Thus, we can conclude that, NLRC3 improves the prognosis of PH by negatively regulating the PI3K-mTOR signaling pathway to suppress the proliferation, migration, and inflammation of PASMCs, NLRC3 may be the therapeutic target, diagnostic index, or prognostic index for PH.

#### 4.4.2 Ventilator-induced lung injury

Ventilator-induced lung injury is very common in clinical practice. Mechanical forces cause lung tissue injury, activate the immune system, and release a large number of cytokines that further destroy lung tissue, eventually lead to more serious clinical manifestations (74). When VILL induced by mechanical ventilation, the expression of NLRC3 in lung tissue of mice was downregulated (75, 76). Both dexmedetomidine and leptin have beneficial roles on the progress of VILI (75, 76). It was observed in the VILI model, the expression of NLRC3 was higher in mice treated with dexmedetomidine or leptin than in the control group (75, 76). Thus, we can speculate that, NLRC3 can alleviate the pathological damage of VILI, however the specific mechanisms involved require further investigation.

#### 4.4.3 Intestinal dysfunction associated with type 2 diabetes

Some patients with Type 2 diabetes (T2DM) with complications such as gut flora dysbiosis, chronic gut inflammation, and increased intestinal permeability. gut flora dysbiosis induces chronic intestinal inflammation, destroys the tight junction of intestinal epithelial cells, and increases intestinal permeability (77).

NLRC3 has beneficial effect on intestinal dysfunction associated with T2DM (78). Overexpression of NLRC3 ameliorated epithelial integrity and up-regulated the expression of tight junction proteins ZO-1 and occludin in colonic epithelial cells (78). In addition, researchers also observed that Butyrate, a short-chain fatty acid (SCFA), binds to the G-protein coupled receptor 43 (GPR43) on the colonic epithelial cell to stimulate the expression of NLRC3 (78).

## 5 Conclusion and future prospects

Based on the finding, we can conclude that NLRC3 is involved in mediating the excessive inflammatory response and uncontrolled cell proliferation. The development and progression of a large number of diseases undergo both pathological process, such as cancer, autoimmune diseases and stroke (**Table 1**). Thus, NLRC3 is a promising molecule and may be an important diagnostic marker and therapeutic target for many diseases. However, how NLRC3 exerts its regulatory activity on intracellular cell signaling has not been thoroughly evaluated; areas requiring further study include NLRC3-mediated mechanisms in the promotion of cell apoptosis, on the differentiation naïve T cells, and regulation of the cell cycle. In addition, the studies of NLRC3 are limited to macrophages, dendritic cells, T cells, and epithelial cells models, however NLRC3 has also been detected in other cells, such as B cells, neutrophils, and basophils, thus, further studies investigating the role of NLRC3 on these cells may be meaningful.

**Table 1:** Roles of NLRC3 in diseases.

Disease	Mechanism or manner	Effect or role	Reference
colorectal cancer (CRC)	NLRC3 inhibits PI3K-AKT-mTOR and PI3K-AKT-FoxO3a/FoxO1 signaling pathway	NLRC3 attenuates tumor cells proliferation, prevents the progress of CRC	(1, 2)
		NLRC3 promotes tumor cells apoptosis, prevents the progress of CRC	(1)
	Bioinformatic analysis	The expression of NLRC3 decreased in colorectal cancer, and the reduction degree was associated with the malignant degree of CRC	(3)
hepatocellular carcinoma (HCC)	NLRC3 attenuates JAK2/STAT3 signaling pathway	NLRC3 prevents cell proliferation, migration, and invasion and promotes cell apoptosis and inhibit the onset and progression of HCC	(4)
	NLRC3 promotes the expression of CCL5 and CXCL9 in tumor issue	The expression of NLRC3 increases the infiltration of CD8+ T cells in tumor tissue, enhances survival in HCC patients.	(5)
	Bioinformatic analysis	Lower expression level of NLRC3 correlates with poor prognosis of HCC	(6–8)
Gastric cancer (GC)	Bioinformatic analysis	NLRC3 expression level negatively correlates with the malignant degree of GC	(9)
lung adenocarcinoma,	Bioinformatic analysis	High expression of NLRC3 is associated with a good prognosis for tumors	(10)
Bladder cancer	NLRC3 attenuates mTOR and Wnt/β-catenin signaling pathways	NLRC3 prevents the proliferation, migration and angiogenesis of tumor cells and inhibits the progression of bladder cancer	(11)
RNA virus	NLRC3 negatively regulates the function of T cells	NLRC3 inhibits the immunity response toward RNA virus	(6, 12)
DNA virus	NLRC3 negatively regulates STING/TBK1 signaling pathway	NLRC3 inhibits the immunity response toward DNA virus	(13)
Gram negative bacillus	NLRC3 negatively regulates NF-κB signaling pathway in a TRAF6-dependent manner.	NLRC3 inhibits the immunity response toward Gram negative bacillus.	(14)
Keratitis caused by Pseudomonas aeruginosa (PA)	NLRC3 negatively regulates NF-κB signal pathway *via* promoting the K48-linked polyubiquitination of IRAK1	NLRC3 attenuates the activation of macrophage and inhibits the excessive inflammatory reaction and protects conceal tissue from injury.	(15)
Mycobacterium tuberculosis infection	NLRC3 negatively regulates NF-κB and ERK/MEK signal pathway	NLRC3 inhibits the activation of CD4+T cells and mediates immune evasion of Mycobacterium tuberculosis	(16)
	NLRC3 inhibits the proliferation and differentiation of CD4+T cells	NLRC3 inhibits the proliferation and differentiation of CD4+T cells and mediates immune evasion of Mycobacterium tuberculosis	(16)
Multiple sclerosis (MS)	NLRC3 negatively regulates NF-κB and ERK/MEK signal pathways, and regulate energy metabolism	NLRC3 prevents the activation of T cells and improves the prognosis of MS	(6)
	NLRC3 prevents naïve T cells from differentiating into Th1 and TH17 cells	NLRC3 improves the prognosis of MS	(6)
	NLRC3 regulates the kind of cytokines production by DC *via* P38 signal pathway, further affects the differentiation of naïve T cells	NLRC3 improves the prognosis of MS	(17)
Alzheimer’s disease (AD)	NLRC3 inhibits PI3K signal pathways	NLRC3 improves learning and memory ability and inhibits the deposition of Aβ, the activation of glial cells and the degeneration of neurons, improving the prognosis of AD	(18)
	Exome-wide rare variant analysis	The rare variants of NLRC3-coding gene had been observed in EMIF-AD	(19)
Ischemia/reperfusion (I/R)	NLRC3 inhibits inflammatory response *via* negatively TRAF6/NF-κB signal pathway.	NLRC3 alleviates nerve injury after cerebral ischemia-reperfusion	(20)
Cutaneous wound healing	NLRC3 inhibits with Hsp90, prevents the ubiquitin of P53, up-regulates the activation of P53 signaling pathway.	P53 signaling pathway inhibits inflammation and cell proliferation, prevents cutaneous wound healing	(21)
Pulmonary hypertension (PH)	NLRC3 negatively regulates PI3K/mTOR signal pathway in pulmonary artery smooth muscle cells (PASMCs)	NLRC3 suppresses the proliferation, migration, and inflammation of PASMCs, thus inhibits pulmonary vascular remodeling, improving the prognosis of PH	(22, 23)
Ventilator-induced lung injury (VILI)		NLRC3 alleviates the pathological damage of VILI	(24, 25)
Intestinal dysfunction associated with Type 2 diabetes (T2DM)	NLRC3 attenuates TRAF6 signal pathway	NLRC3 ameliorates epithelial integrity and up-regulates the expression tight junction proteins ZO-1 and occludin in colonic epithelial cell	(26)

NLRC3 as a member of the PRR family, its function in pathogen recognition has been addressed by some researchers. In contrast to many other NLRs, the LRR domain of NLRC3—which recognizes pathogens—is positively charged. This is an interesting discovery, as numerous antigens are negatively charged, which means that NLRC3 might have the ability to recognize antigens. Further research is needed to confirm this speculation.

In conclusion, the proven functions of NLRC3 are preventing the activation of the immune system, inhibiting the inflammatory response, attenuating cell proliferation, and promoting apoptosis. In addition, the roles and application of NLRC3 in specific disease processes, requires further insight and exploration on how to translate mechanism research to a direct clinical application. In addition, we speculate that NLRC3 may have the function of recognizing antigens, whether this hypothesis is true, and what type of antigens NLRC3 can recognize, warrants further research.

## Author contributions

JX and YS designed the review. DS and JX wrote the manuscript with supervision of YS. All authors critically revised the manuscript and approved it for publication.

## Funding

This study was supported by National Natural Science Foundation of China (No. 81772047, 81971818, 82002026 and 82272217) and the National Key Research and Development Project (2021YFC2500802).

## Acknowledgments

Thanks for researchers who contribute to the exploration of NLRC3.

## Conflict of interest

The authors declare that the research was conducted in the absence of any commercial or financial relationships that could be construed as a potential conflict of interest.

## Publisher’s note

All claims expressed in this article are solely those of the authors and do not necessarily represent those of their affiliated organizations, or those of the publisher, the editors and the reviewers. Any product that may be evaluated in this article, or claim that may be made by its manufacturer, is not guaranteed or endorsed by the publisher.
